# Selective targeting of p53 gain-of-function mutants in cancer

**DOI:** 10.18632/oncoscience.408

**Published:** 2018-04-29

**Authors:** Achuth Padmanabhan

**Affiliations:** Department of Molecular and Cellular Biology, Baylor College of Medicine, Houston, TX. 77030, Dan L Duncan Comprehensive Cancer Center, Baylor College of Medicine, Houston, TX. 77030, Center for Reproductive Medicine, Baylor College of Medicine, Houston, TX. 77030

**Keywords:** mutant p53, cancer, personalized therapeutics, p53, USP15

**Figure 1:**
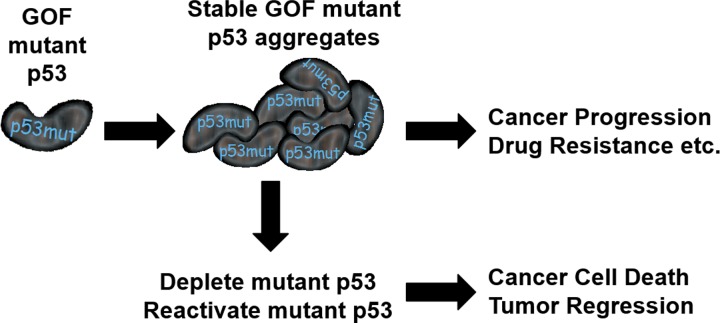
GOF mutant p53 proteins form stable aggregates in cells and promotes tumor progression and development of drug resistance Depletion of mutant p53 or their reactivation induces cancer cell death and causes tumor regression.

Gain-of-function (GOF) mutations in *p53* are frequent in many human cancers and play key roles in tumor progression and development of drug resistance [1] (Figure 1). Unlike tumor suppressive wild-type (WT) p53 protein, which is rapidly turned over in cells by the ubiquitin proteasome system, the GOF mutants form stable aggregates that accumulate in cancer cells [1, 2] (Figure 1). Depletion of mutant GOF p53 mutants in cancer cells has been shown to induce cancer cell death; demonstrating a key role for these mutants in cancer cell survival and tumor progression [3, 4]. While the therapeutic merits of strategies that can selectively deplete GOF mutant p53 proteins in cancer cells are well appreciated, achieving such selective depletion in a clinically translatable manner has been difficult. A major challenge in developing a clinically viable strategy to selectively target mutant p53 proteins in cancer has been the need to differentiate and exclude the tumor suppressive wild-type p53 protein present in the healthy cells from being targeted. Realizing this goal requires better understanding of upstream regulators and pathways that selectively regulate the different p53 GOF mutants in cells. Recently, the deubiquitinase USP15 was identified as a selective upstream regulator of the p53- R175H conformational mutant in ovarian cancer cells [3]. Depletion of USP15 in ovarian cancer cells causes decrease in p53-R175H protein levels and induces cell death in cancer cells expressing this mutant form of p53 [3]. Thus, targeting USP15 provides a new and selective way to deplete p53-R175H protein and achieve killing of cancer cells carrying this mutation. USP15 levels have been shown to be elevated in many cancers and several new evidences linking the role of this deubiquitinase to cancer progression is beginning to emerge [5]. In addition to identifying a selective druggable regulator of p53-R175H mutant, this discovery also established the existence of unique regulators of the different GOF p53 mutants. Identifying these regulators will open up new avenues to target the respective oncogenic mutations in cancer cells.

Other innovative approaches being pursued currently to target the GOF p53 mutants in cancer cells includes identifying small molecules and peptides that can bind to the mutant p53 proteins and induce conformational changes in the mutant protein that converts it to a more WT-like conformation [6]. This process is known as ‘reactivation' [6]. Reactivation thus helps increase WT p53 levels in cancer cells, which would in turn cause tumor suppression and activation of apoptosis in cancer cells [6]. Further, by converting the stable GOF p53 mutants to WT p53-like proteins, these small molecules will enable detection of mutant p53 by the targeted protein turnover mechanisms in cells that regulates the degradation of WT p53, leading to their depletion. Several pharmacological molecules have been identified so far that can cause reactivation of mutant p53. These include PRIMA-1 (p53 Reactivation and Induction of Massive Apoptosis), MIRA-1 (Mutant p53 reactivation and Induction of Rapid Apoptosis), and the methylated analog of PRIMA-1 (PRIMA-1MET or APR-246) [6]. While, the mechanisms through which these small molecules act differ, they have all enjoyed considerable success during *in vitro* experiments and pre-clinical animal studies. APR- 246 is currently being tested in phase II clinical trials for platinum resistant high-grade serous ovarian cancer with mutated p53 [7].

Despite the promise of the current batch of mutant p53 targeting therapeutics, it is important for us to continue our efforts to identify new ways to selectively target mutant p53 in cancer cells as the extant molecules could face challenges during their clinical translation or have issues with development of drug resistance upon prolonged use. Pharmacological targeting of mutant p53 by small molecules is a rapidly evolving field that holds tremendous promise and potential to pave the way towards the development of novel anti-cancer agents that would allow personalized treatment based on the p53 mutation status of the patient's tumor. Understanding how the various GOF mutant forms of p53 differ, their specific roles in cancer progression, and developing novel therapeutics and strategies to target them selectively should certainly be priorities worth investing going forward.
